# Behavioral responses of the European mink in the face of different threats: conspecific competitors, predators, and anthropic disturbances

**DOI:** 10.1038/s41598-021-87905-5

**Published:** 2021-04-15

**Authors:** Lorena Ortiz-Jiménez, Carlos Iglesias-Merchan, Isabel Barja

**Affiliations:** 1 Department of Biology, Zoology Unit, Universidad Autónoma de Madrid, Madrid, Spain; 2 Department of Forest and Environmental Engineering and Management, Universidad Politécnica de Madrid, Madrid, Spain; 3 Biodiversity and Global Change Research Center (CIBC-UAM), Universidad Autónoma de Madrid, Madrid, Spain

**Keywords:** Animal behaviour, Conservation biology, Behavioural ecology

## Abstract

Prey species assess the risk of threat using visual, olfactory, and acoustic cues from their habitat. Thus, they modify their behavior in order to avoid encounters with competitors, predators, and human disturbances that endanger their fitness. European mink (*Mustela lutreola*) is a critically endangered species that can be preyed upon by larger carnivores and displaced by dominant conspecifics to areas of lower quality, e.g., near to more anthropized localities which may be noisier. In this study, the behavioral responses of 24 European mink were evaluated by conducting an experiment in which the presence of a conspecific competitor was simulated with a visual cue (mirror) and the presence of predators (terrestrial and aerial) with odorous cues. Additionally, they were also exposed to potential sources of anthropic disturbance with acoustic cues (road traffic noise and human voices). Our results showed that European mink were hidden for longer periods of time due to the presence of conspecifics and being exposed to the fecal odors of a terrestrial predator such as dog, but especially when they were exposed to anthropic noises. In the presence of a conspecific, the females and the subadults were the ones who remained hidden for the longest time. As well, they were hidden for longer periods of time due to the presence of conspecifics but in combination with dog feces and anthropic sounds did not induce variations in the response, as both by themselves already triggered an increase in the time they spent hiding. The vigilance model showed the effects of the same factors as the hiding model, but with antagonistic effects in the case of vigilance time which decreased during anthropic noises exposition. Finally, we want to highlight that European mink showed an innate response favorable to all three types of threats, but attention should be focused on human disturbances—as they trigger the most extreme responses—which may affect the rate of survival of this threatened species.

## Introduction

The fitness of a species depends largely on its ability to detect threats through direct (visual) or indirect (olfactory and acoustic) cues from their environment^1–3^. These cues alert them to the presence of competitors, predators, and anthropic disturbances^4^. Thus, animals adopt different strategies that affect their ecology, either altering their behavior or/and triggering a stress response^5, 6^. The most appropriate strategy is to assess the risk of threat, and the costs and benefits it brings in terms of survival, reproduction, or feeding time^7^.

Early detection of competitors allows animals to reduce the cost of defending resources^8^. It has been shown that many mammals, especially carnivores, use chemical communication to obtain information about the reproductive and social status of a conspecific, as well as intra-sexual competition (territorial defense and choice of partner)^9, 10^. For instance, mustelid species use feces, urine, and glandular secretions which act as visual and aromatic markers^11, 12^.

In addition, when feces are used not only as an olfactory cue but also as a visual cue, animals deposit them in substrates and areas that improve their detection by specific species and their persistence in time^10, 13^. In this way, animals reduce the likelihood of direct and unexpected encounters with a competitor, at which point the most common responses are usually those that are triggered more quickly such as confrontation (aggressiveness) or flight (hiding)^14, 15^. Predation is a type of biological interaction that promotes the maintenance of equilibrium in the ecosystem and plays a key role in natural selection^16^. On the one hand, predation is an ecological factor that affects prey abundance and, on the other hand, affects the variety of prey adaptations to avoid being detected, captured, and consumed by their predators^17^. Several studies showed that the alteration of the small mammals population densities may be due to a modification of their behavior after detecting a predator: decreased of foraging and feeding, interruption of reproductive activity, and restriction of habitat use^18–21^. These behavioral alterations entailed a compromise between the performance of anti-predatory behaviors and non-defensive behaviors^21–24^.

Mammals develop different anti-predator strategies (immediate or delayed) depending upon the type of predator to which they are exposed^25^. Thus, research work on gibbons (*Hylobates lar*) found that individuals discriminated against their potential predators basing decisions on their silhouette and morphology. The territory marks such as olfactory cues (e.g., volatile compounds in their excreta, urine, and anal secretions) used by predators to communicate with their conspecific are considered indirect signals which, at the same time, may alert their presence to prey species^10, 26^. Furthermore, vocalizations emitted by a predator are also a type of indirect signal that prey can use to assess the risk of predation. A study of two sympatric gull species (*Larus marinus* and *Larus argentatus*) showed that individuals discriminated between different auditory threat indicators and responded proportionately to the perceived threat level^27^.

Although most of the acoustic cues studied belong to vocalization of potential natural predators, there are recent studies that point to the potential negative impact that anthropic noise pollution (in particular, road traffic noise) may have on biodiversity^28^. In fact, it is known that gene flow patterns can be altered by the proximity of populations to the road, which can produce a genetic isolation that results in gene drift and inbreeding^29–31^. Numerous studies showed how noise pollution may affect the behavioral and physiological responses of animals^31–33^. Apart from transport infrastructures, leisure activities may also cause negative impacts on nature. Tourism in protected areas is on an increasing trend for decades now^34, 35^: several studies have shown how human activities triggered an increase in the physiological stress response in vertebrates, often resulting in pathological consequences^36–38^. Furthermore, it has been observed that animals may perceive humans as potential predators^39^, as anti-predatory responses have been recorded based on the transfer of populations to areas free of disturbance but of worse quality^38, 40^ and a change in the distribution of time for each behavior^41, 42^.

Many studies show the negative effects of roads sound and human activities or infrastructures on vertebrates^31, 32^. However, it is not known whether both noises are equally disturbing. So, our research could provide new data for the conservation of species in situ where both noises are quite common due to ecotourism, especially in Natural Parks. In addition, it is of high interest for critically endangered species to inhabit these protected areas to promote their conservation. Consequently, evaluating three types of potential threat (conspecific competitor, predator and anthropic noises) from the environment and, at the same time, three different types of sensory (visual, olfactory and acoustic) signals allows for a broader view of the anti-predatory response of any species. However, since in the natural environment these signals are not only manifested separately, combining different types of cues (visual, olfactory and acoustic) provides added value to studies in animal behavior, especially if they are in a vulnerable situation.

European mink is the most threatened mustelid in Europe^43^. Currently, there are only three stable populations (in Russia, in the Danube Delta, and in Spain/France) which are very fragmented^44^. Furthermore, given their low reproductive rate, their conservation is now seriously compromised^45^. In addition, no studies are published on how these animals respond to threats from their environment. In fact, the interaction with conspecifics is little studied, although everything suggests that they communicate chemically like the rest of the mustelids^46^. European mink can be preyed upon by other carnivores of both aerial (raptors) and terrestrial (foxes and domestics dogs)^47, 48^ types and mink can also act as a predator and prey depending on the circumstances. This species is a nocturnal and twilight^49^ so the most developed and used sense, both in intraspecific and interspecific communication, is smell as in other carnivores^50^ The visual, olfactory and auditory senses are well developed in mustelids; however, vision is not as important for them as another mammals such as primates^51^.

This research stems from the need to know the implications of natural and anthropic factors from the environment in the conservation of European mink.

Our aim was to understand how different signals of threat from conspecific competitors, predators, and human disturbances modify behavioral responses. In addition, our particular focus was on the effect of the combination of a visual cue with one of the other two modes of cues (olfactory and acoustic) to observe the implications of the presence of a conspecific species during a predation situation and an anthropic threat situation.

Our hypotheses were that: (i) the presence of conspecifics (visual cue) would increase time spent in the nest box to avoid confrontations due to inter and intrasexual competition^52^ and decrease the vigilance time since more time spent hiding in the nest box allow the individuals not to waste energy on being vigilant^53^; (ii) odors from predators (olfactory cues) and anthropic noise (acoustic cues) would trigger an anti-predatory response as in increased time spent in the next box (avoidance using a refuge)^54^ and increased time spent on vigilance^55^; (iii) time spent in the nest box and vigilance time would differ between the two types of predators (owl and dog) since the dog is more easily recognized as a predator due to the volatile components of its excrement derived by a carnivore diet^56^; and between the two types of noises (road and human voices) due to the characteristics of both sounds.

## Materials and methods

### Study subjects and enclosures

The study was conducted on 24 European mink: four subadult males, seven subadult females, six adult males and seven subadult females. These European minks were born in captivity and are part of an ex situ breeding and reintroduction plans into the natural habitat. The animals were housed individually in pens belonging to the Foundation for Research in Ethology and Biodiversity (FIEB), a breeding center located in Casarrubios del Monte (Toledo, Spain). The enclosures had a surface area between 40 and 60 m^2^ and were naturalized with riparian vegetation and individual pool of 5 × 3 × 0.70 m deep. The access to the enclosures was via a covered corridor where each individual’s nest boxes are located, and a window for each enclosure designed to minimize animal-caretaker contact during food supply and observational studies. European mink were fed on raw trout, raw quail, raw chicken, cooked egg, live mice, and live rats once a day in the afternoon. The amount of food supplied varied according to the day depending on the animal’s last recorded weight and the calorie index of each food item.

### Experimental design and behavioral data collection

The experiment consisted of placing three types of cues into each enclosure to simulate the presence of conspecific (visual cues), predators (olfactory cues), and human disturbances (acoustic cues). No individual was ever exposed to any of those cues before. The experiment lasted 30 days (from January to February) and it was divided into two phases of 15 days each, phase 1 with the absence of a conspecific and phase 2 with the presence of a conspecific. During each phase, European mink were exposed to three consecutive days without olfactory or acoustic cues (control treatment) and four different treatments (three consecutive days each one): freshly collected owl feces, fresh dog feces (olfactory cues, 7 g/enclosure of each), road traffic noise and community noises caused by human voices (acoustic cues, 4 min each). European minks were exposed to all conditions (control and all three types of cues) pseudo randomly across individuals. Ambient sound level (background sound pressure level or background noise) at each enclosure during the control treatment was measured.

European mink behaviors were recorded during four minutes through the feed window by researcher using a SONY Xperia Z5 mobile phone in HD quality. The start of the daily test began when hatch of the nest box was opened, and it finished when the 4 min elapsed. Since we previously observed that the European mink was a very active animal that performed numerous behaviors in a short time, other studies with mammals that use a few minutes were considered^57–59^. Moreover, we observed previously that the European mink is a very fast, active and agile animal when it is out of the burrow, so four minutes are enough to obtain an appropriate volume of data. In addition, assessing the behavior of a longer-lasting cue could lead to habituation and causes bias in the data, especially in odorous cues which its volatiles are quickly lost.

The recorded behaviors were transcribed into a data matrix using an individual focal sampling (sampling type) with a one-zero-time record (recording type) with 10 s-intervals for a sample of 24 European minks. Individual focal sampling consists of observing an individual for a certain time (in this study 4 min). One-zero-time record is used to record whether the behavior of interest has occurred within each 10 s-interval in which the 4 min were divided^60^. The behaviors recorded were time spent in the nest box (s) and vigilance time (s). Time spent in the nest box was defined as the time (s) that each individual stayed inside their nest box. Vigilance time behavior (s) included the sum of the following behaviors: resting guarding (the individual exhibited the head or remained with the rest of the body hidden in his hiding place), quadrupedal guarding (the individual contemplated and examined somewhere in their enclosure pausing on all four legs) and bipedal guarding (the individual scrutinized the enclosure by positioning only on his hind legs in a vertical position or leaning his front legs on a solid surface to help him stay upright)^61^.

### Visual cues

A round mirror (20.3 × 20.3 × 1.5 cm) with two faces, one of them with magnification in which the individuals were reflected, was used in order to simulate the presence of conspecific. The mirror was placed sideways one meter from the exit hole of the nest box within each enclosure using an integrated support. In this way, the minks could only see the edge of the mirror and therefore an object, from the nest box. When approaching, the minks could encounter one side or the other of the mirror depending on the direction they took when exploring the enclosure. We simulated the presence of a conspecific at different distances, one closer and the other more distant depending on the magnification of the mirror. We previously tested in another study that minks did not present neophobia to the mirror or changes in time spent in the nest box and vigilance time behavior due to the mirror face where they were reflected (Ortiz-Jiménez et al. unpublished data).

### Olfactory cues

Olfactory cues were simulated with feces of two potential predators: Eurasian eagle owl (*Bubo bubo*) and dog (*Canis lupus familiaris*)*.* Feces were collected from adult owls of both sexes and from dogs that inhabit in FIEB to ensure that they were de-wormed. It was possible to differentiate fresh feces because of the presence of a mucus layer, without signs of dehydration, and of strong odor^20, 36^. These feces were frozen at -20ºC until required for the experiments. All frozen fresh-collected owl feces were mixed and homogenized to avoid bias, just like the dog feces, since volatile compounds can vary depending on the individual, sex, age, and season^62–64^, even depending on the amount of volatile concentration in each excrement^65^. During the experiment, feces were placed one meter from the exit hole of the nest box within each enclosure in the morning. Feces were removed at the end of each trial and were replaced by new fresh feces at the beginning of a new trial. When the feces were removed, the substrate under them was collected so that no odor remained. This was verified by observing that the minks did not specifically return to this site after excrement removal.

### Acoustic cues and spectral characteristics

A playback experiment was conducted to simulate human disturbances using two pre-recorded signals, road traffic noise and human voices. The first audio file corresponded to road traffic noise signal which was recorded from a bike bridge located in highway M-604 in Madrid. Traffic density was estimated in approximately 3700 vehicle per hour and the average speed was limited to 80 km/h at the recording site location. The second audio file corresponded to community noise which was composed of loud human voices recorded next to a school playground.

**Figure 1:**
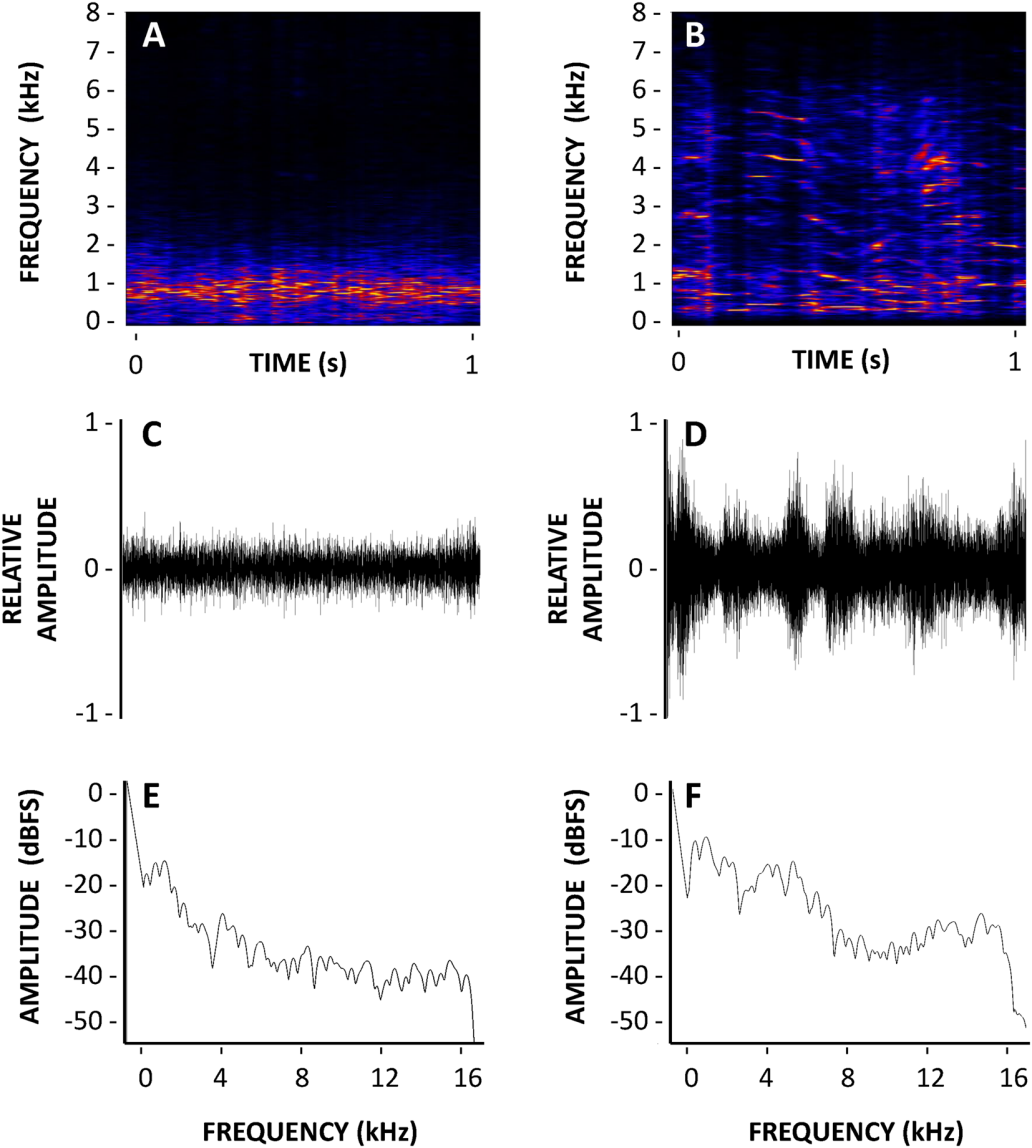
(**A**) Spectrogram illustrating the variation of frequency and intensity of the road traffic noise cue over time. The brighter the color, the more energy is concentrated at that frequency. (**B**) Spectrogram of the human voices cue. (**C**) Oscillogram representing the waveform and relative amplitude of the road traffic noise cue. (**D**) Oscillogram of the human voices cue. (**E**) Power spectra of the road traffic noise cue showing a dominant peak at a frequency of approximately 1 kHz. (**F**) Power spectra of the human voices cue showing a series of peaks at several frequencies.

Figure 1 shows the main characteristics of the acoustic cues which were released. In terms of energy, most of the road traffic noise energy was comprised between 0 and 2.5 kHz (Fig. 1A) and human voices expanded between 0 and 6 kHz (Fig. 1B). Waveform of road traffic noise (Fig. 1C), in which enveloping profile was a relatively flat curve in the time domain, was lower in fluctuations than the shape of the waveform of human voices (Fig. 1D). In addition, the corresponding amplitude-frequency spectrum showed the road traffic noise was characterized by a unique dominant spectral peak at 1 kHz (Fig. 1E). However, two spectral peaks centered at 0.5 kHz and 1 kHz when the audio file corresponding to human voices was released. In addition, human voice signals also showed two secondary peaks which were located at 2.5 kHz and 4 kHz (Fig. 1F).

Finally, sound power level (PWL) of both noise sources was adjusted to approximately 80 ± 0.5 dB (A). It must be noted that PWL is the inherent noise of the device and it does not vary with distance from the noise source. PWL must not be confused with sound pressure level (see below). Recorders were played using a SONY-branded loudspeaker (Personal Audio System SRS-XB2) connected to a SONY-branded digital voice recorder (Digital Dictation Machine ICD-PX370). The playback system was placed in the center of the enclosure, hanging from the ceiling at approximately 1.60 m above the ground level.

### Noise measurements and spectral analysis

Sound pressure level (SPL) for a receiver varied with its distance from a noise source and other factors such as frequency composition, ground absorption, reflecting surfaces or weather conditions. SPL measurements were measured with a professional sound level meter CESVA SC 420 class 1 which was calibrated (calibrators provide a 94 dB signal at the frequency of 1 kHz) before and after each measurement. A sound level meter was held by hand from the feed window at a distance of approximately 1.5 m from the loudspeaker for SPL measurements. The total equivalent noise level in second intervals was recorded, as well as equivalent noise level by 1/1 octaves of the frequency spectrum. Indeed, it is necessary to analyze not only SPL but also the spectral composition of the sound acoustic environment during the experiment. The frequency spectrum of field noise measurements ranged from 16 to 16,000 Hz. The sound level meter measured all the functions simultaneously with all frequency weightings (including A and Z weighting). It is also important to note that A-frequency-weighting is the most commonly used frequency weighting to assess environmental noise. However, it describes sound pressure according to the subjective and nonlinearly response of the human ear. Therefore, given the characteristics of the experiment, we decided to extracteZ-weighting SPL values for the octave bands instead of A-weighting SPL values. The ‘Z’ (Zero) frequency weighting offers a flat frequency response between 10 Hz and 20 kHz according to specifications given in IEC 61672-1:2013. This is an international standard referring to sound level meters. In addition, spectrograms and frequency profile analyses were also carried out for both audio files using Sonic Visualiser version 4.0.1 (Queen Mary University of London, London, UK) and version 2.2.0 of Audacity (Audacity Team) recording and editing software.

### Ethical statement

FIEB Foundation in which the study present was carried out is registered as a zoo center and animal experimentation center covered by Consejería de Agricultura y Servicios Periféricos de Castilla la Mancha with registration code: ES450410000053. This registration carries the implications of housing and handling animals according to animal welfare criteria. Furthermore, FIEB is a participating center in Ex situ Conservation Program for European mink acting as a breeding and research center promoted by Ministerio de Transición Ecológica y Reto Demográfico of Spain.

Since this project is part of the Conservation Program do not need to pass through ethics as it is not invasive. In addition, the stressors put on the minks were those that they experience in natural environment (scent of predators and anthropic noises) and the authors kept them to a minimal when carrying out the study.

### Statistical analyses

Behavioral responses were analyzed using two Poisson distribution Generalized Linear Mixed Models (GLMMs) since the recording of the behaviors was done by a count (seconds in multiples of ten: 1 time equal to 10 s, 2 times equal to 20 s…). The response variables considered in each model were time spent in the nest box (s) and time of vigilance (s). In the GLMMs we included sex (male/female), age (subadult/adult), phase (presence/absence of a conspecific), and treatment (owl feces/dog feces/road traffic noise/human voices) as fixed factors and each individual mink as the random factor. We also tested the effect of the following interactions in the same models: sex*phase, sex*treatment, age*phase, age*treatment, and phase*treatment. Results were considered significant at a probability value of α < 0.05. We used the software SPSS 23.0 for Windows (SPSS Inc, Chicago, IL, USA) for statistical analysis. Data are presented as mean ± standard error.

## Results

### Acoustic cues analysis

Background sound pressure level at each enclosure during the control treatment was approximately 58 dB(Z). Background noise level also deserves to be noted in decibels A (35 dB(A)) to acquire better insight of the scenario. Measurement of the equivalent continuous sound pressure level, Leq (in decibels Z), of acoustic cues was approximately 68 dB(Z) during both playback treatments (i.e., road traffic noise and human voices). However, differences were found in their spectral compositions. Road traffic noise SPL were higher than those of human voices below the octave band of 1 kHz (Table 1). A maximum difference of almost 9 dB was reached at a frequency of approximately 0.125 kHz. Meanwhile, SPL due to human voices resulted in higher values than road traffic noise at every octave band above 1 kHz, and a maximum difference of approximately 12 and 8 dB (in favor of the human voices cue) were found at frequencies of 4 and 8 kHz, respectively.

**Table 1:** Frequency spectra (in octave bands), equivalent continuous sound pressure level measurement and peak level (in dB(Z)) during each playback treatment (4-min long each one).

Frequency (Hz)	16	31.5	63	125	250	500	1 k	2 k	4 k	8 k	16 k	Leq	Peak
Road noise	57.7	54.1	51.2	55.2	59.9	62.1	63.4	53.4	45.5	34.5	18.7	68.4	88.7
Human voices	49.6	48.5	49.1	46.6	58.6	64.1	63.2	56.4	57.4	42.8	22.9	68.1	86.1

### Time spent in the nest box

GLMM results indicated that the time spent in the nest box by mink was explained by the pure effect of age, phase and treatment factors, as well as by the interactions of sex*phase, sex*treatment, age*phase, age*treatment, and phase*treatment. However, the sex factor was not significant in the model (Table 2). Subadult European mink (210 ± 3.53 s) spent more time in their nest box than adults (169 ± 4.34 s). In relation to phase, European mink spent more time in the nest box in the presence of a conspecific (presence: 201.62 ± 3.64 s; absence: 174.58 ± 4.54 s). Finally, regarding the pure effect of the treatment variable time spent in the nest box was longer during the noise treatments (traffic noise: 217.64 ± 4.15 s; human voices: 216.88 ± 4.78 s) than during the odor treatments (dog feces: 192.08 ± 5.68 s; owl feces: 172.01 ± 6.78 s) and during the control (138.38 ± 8.63 s).

**Table 2:** Results of the GLMM using time spent in nest box (s) of European mink as a response variable analyzing the pure effect of the factors (sex, age, phase and treatment) and their interactions (sex*phase, sex*treatment, age*phase, age*treatment and phase*treatment).

Factor	*F*	*df1*	*df2*	*p*
Intercept	258.39	21	451	**0.001**
Sex	0.02	1	44	0.882
Age	6.69	1	44	**0.013**
Phase	1039.54	1	690	**0.001**
Treatment	905.85	4	690	**0.001**
Sex*phase	20.37	1	690	**0.001**
Sex*treatment	61.38	4	690	**0.001**
Age*phase	5.704	1	690	**0.017**
Age*treatment	75.21	4	690	**0.001**
Phase*treatment	476.38	4	690	**0.001**

**Figure 2:**
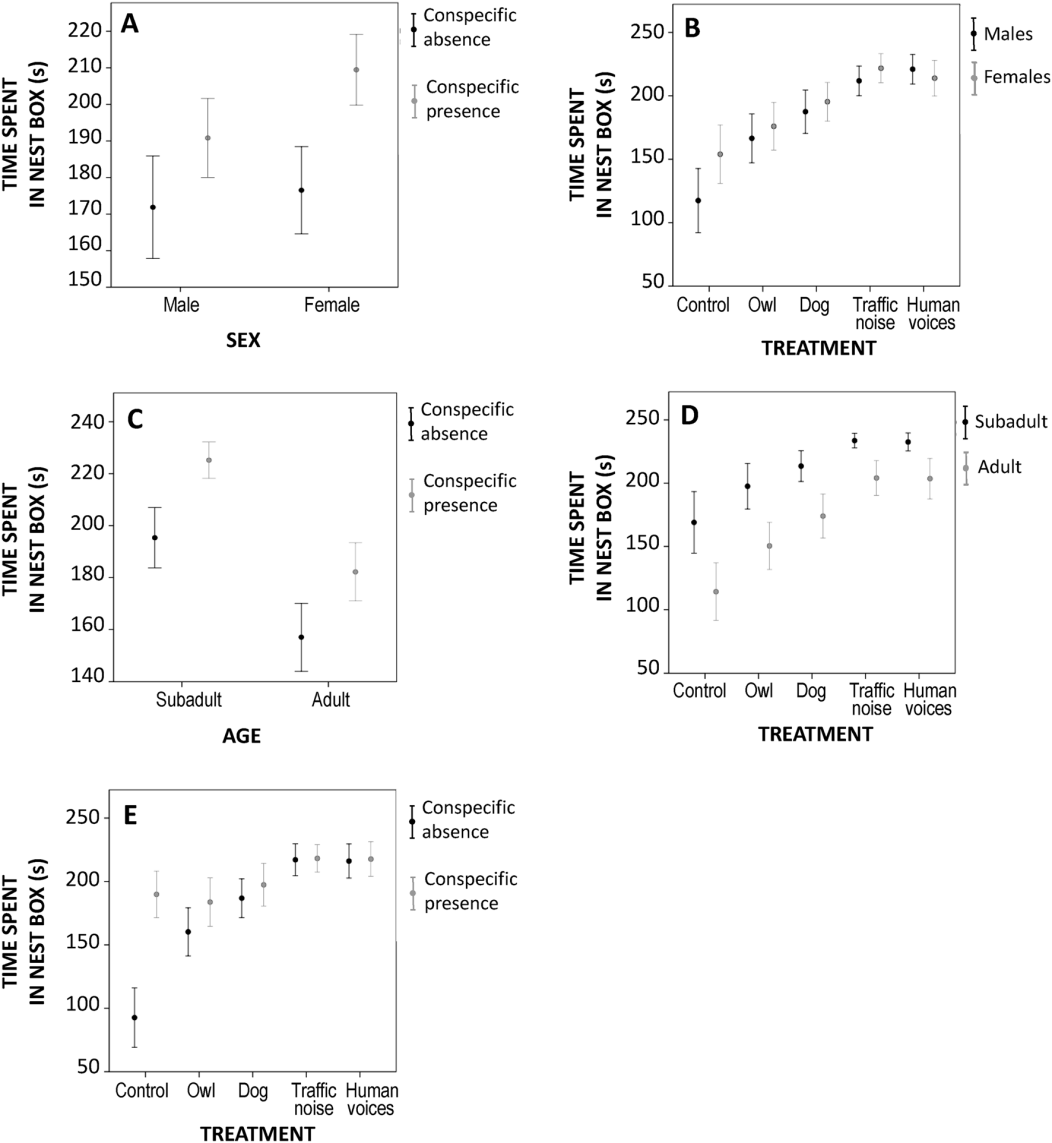
Mean time spent in the nest box (s) ± SE according to (**A**) sex*phase (**B**) sex*treatment (**C**) age*phase (**D**) age*treatment and (**E**) phase*treatment.

The 2-ways interaction between sex and phase showed that the presence of a conspecific increased the time spent in the next box in both sexes, being more pronounced in females (Fig. 2A). The interaction between sex and treatment showed that both, males, and females spent more time in their nest box when they were exposed to anthropic noises than predator odors (Fig. 2B). Moreover, the interaction between age and phase showed that the response was more pronounced in the presence of a conspecific, showing the highest differences in the case of subadults (Fig. 2C). The interaction between age and treatment showed that time spent in nest box in both ages was increased during odor treatments and road traffic noise, there being no significant differences over time in the nest box during both types of sounds (Fig. 2D). Furthermore, the interaction between phase and treatment showed that European mink spent more time in their nest boxes when they were exposed to the control and the owl feces treatments than when they were exposed to the other treatments in the presence of a conspecific. Nevertheless, during the dog feces treatments and the noise treatments, there was no difference both in the presence and in the absence of a conspecific (Fig. 2E).

### Vigilance

The GLMM for vigilance indicated that the time spent on this behavior was explained by phase, treatment, and the following interactions: sex*phase, sex*treatment, age*phase, age*treatment, and phase*treatment (Table 3). European mink conducted vigilance for less time during the human voices treatments (71.94 ± 8.03 s) compared to road traffic noise (135.21 ± 11.56 s), odor treatments (dog feces: 155.76 ± 11.24 s; owl feces: 146.25 ± 11.19 s) and control (131.76 ± 10.80 s). European mink decreased the time spent on vigilance in presence of a conspecific (presence: 104.52 ± 6.23 s; absence: 151.25 ± 7.28 s).

**Table 3:** Results of the GLMM using vigilance time (s) of European mink as a response variable analyzing the pure effect of the factors (sex, age, phase and treatment) and their interactions (sex*phase, sex*treatment, age*phase, age*treatment and phase*treatment).

Factor	*F*	*df1*	*df2*	*p*
Intercept	701.9	21	689	**0.001**
Sex	1.43	1	689	0.233
Age	2.55	1	689	0.111
Phase	2807.9	1	689	**0.001**
Treatment	1031.75	4	689	**0.001**
Sex*phase	739.81	1	689	**0.001**
Sex*treatment	380.49	4	689	**0.001**
Age*phase	586.52	1	689	**0.001**
Age*treatment	347.01	4	689	**0.001**
Phase*treatment	1064.94	4	689	**0.001**

**Figure 3:**
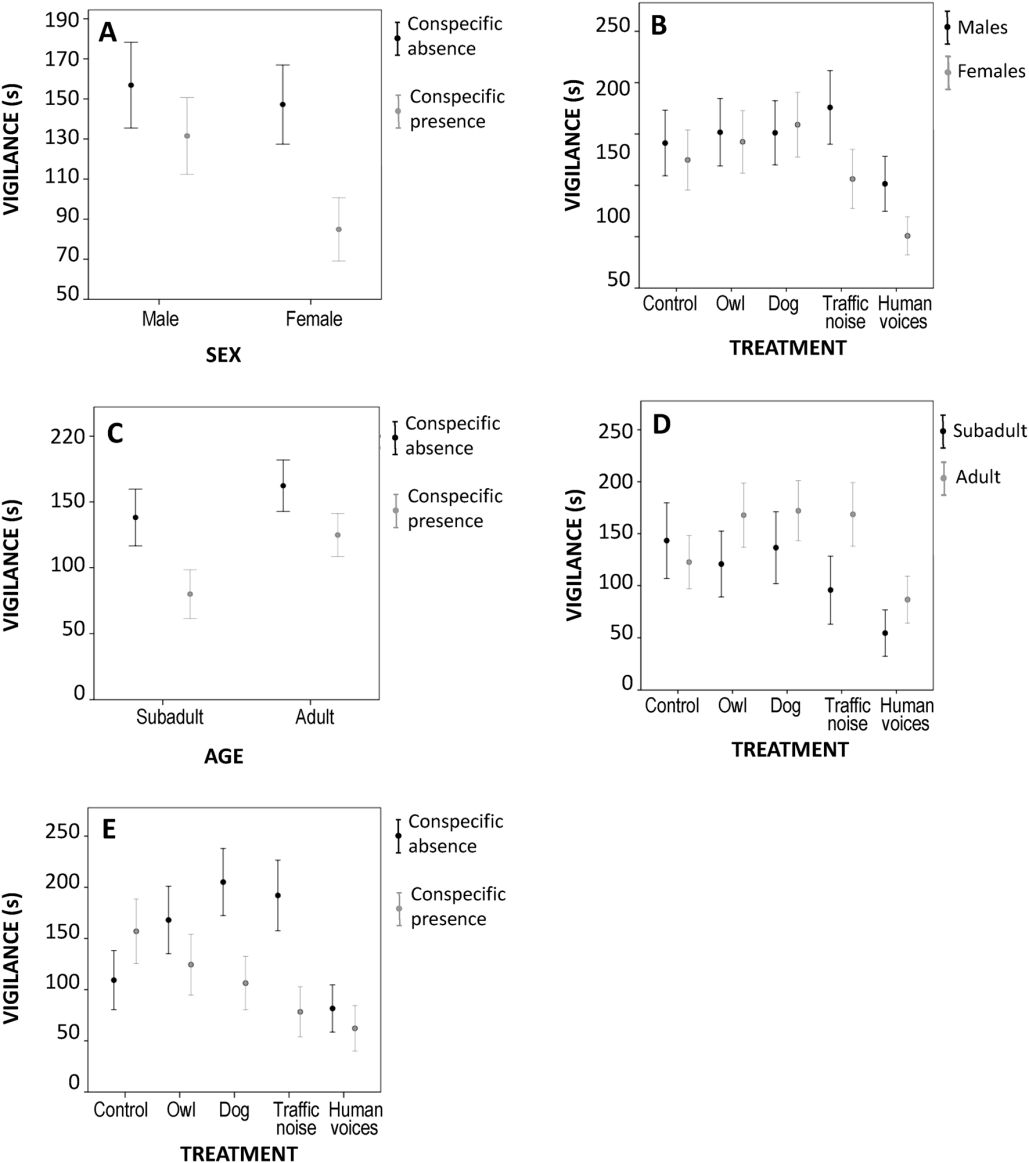
Mean time spent on vigilance (s) ± SE according to (**A**) sex*phase, (**B**) sex*treatment) age*phase, (**D**) age*treatment and (**E**) phase*treatment.

The 2-ways interaction between sex and phase showed that both sexes decreased the time they spent on vigilance in the presence of a conspecific, especially the females (Fig. 3A). The interaction between sex and treatment showed that females spent less time on vigilance during the noise treatments, especially during the human voices, while males only decreased their time on vigilance during human voices (Fig. 3B). Moreover, the interaction between age and phase showed that both ages decreased their vigilance time in the presence of a conspecific, especially subadults (Fig. 3C). The interaction between age and treatment showed that subadults spent less time on vigilance during the noise treatments, especially during human voices, while adults decreased their time on vigilance only during human voices (Fig. 3D). Ultimately, the interaction between phase and treatment showed that individuals spent less time on vigilance when they were exposed to predator odors and road traffic noise. Note that there was no difference in the time of vigilance during human voices whether there is a conspecific or not (Fig. 3E).

## Discussion

Our major findings were: (1) the presence of conspecifics in itself increased the time in the nest box except when this condition arose at the same time as the dog odor and anthropic sounds, which themselves have already induced this response; (2) the presence of conspecifics in itself decreased the time spent on vigilance except when this condition took place at the same time as the noise of human voices that already triggered this response; (3) the European minks developed an adequate innate anti-predatory response based on the avoidance of predators using the nest box for longer; (4) anthropic sounds were the ones that triggered an increase in the time most pronounced in the nest box, being the human voices that most influence the reduction of the vigilance time.

### Presence of a conspecific

In general, European mink increased their time in the nest boxes and decreased their time on vigilance in relation to the presence of a conspecific. Presumably, European mink interpret the presence of a competitor as an unexpected and surprised threat from a direct visual cue because there was previously no odor trace to warn them of competitor presence. Since the individuals were not prepared for an unanticipated confrontation, they chose a flight response that it was manifested in a longer time in hiding. The modification of responses could derive from a large-scale behavioral adjustment to reduce the risk of encounter in the same space and time with a larger or more dominant conspecific competitor^66^. However, as there are hardly any published studies on the vision of the European mink, it is possible that minks simply get scared with their own reflection without being perceived itself as a conspecific because their sense of smell is the most developed, so they might not know how to identify another mink without the support of the smell. As well, although it was preliminarily tested that there was no neophobia caused by the mirror, not being able to control the visual capacity of individuals could be a limitation of this study.

In addition, we observed behavioral differences depending on sex of the individuals in the presence of a conspecific. Females spent more time in their nest boxes and decreased their time on vigilance in a more pronounced way than males, probably due to intersexual competition. A research about American mink (*Neovison vison*) determined that the females avoided, as far as possible, coinciding with other mink to avoid forced encounters with larger and more dominant males during the non-breeding season^52^. Furthermore, the vigilance time of the males did not decrease as much as that of the females probably due to their territorial character, as also suggested by some studies with ermine *(Mustela erminea*)^67^ and badger (*Meles meles*)^68–70^, which showed that the males were defending more extensive territories than the females by assessing more thoroughly the evidence of a conspecific competitor. Furthermore, we observed that the subadults were who spent more time in the nest box and decreased the time spent on vigilance more markedly. Since subadults are not sexually mature^49^, it probably compensates as an evasive rather than territorial attitude with conspecifics. Finally, the presence of a conspecific increased the time spent in the nest box when this condition occurred at the same time as the presence of feces from an owl compared to control without mirror. This is probably due to the fact that the mink assessed the threat of predation by owl as a lower risk than per dog, so that the additional presence of a mirror (direct signal from a specific conspecific) boosted the increase of time spent in the nest box. McCormik and Manassa^71^ determined that information to assess the risk of predation had a benefit when different natural signals such as visual and olfactory cues were combined. However, the presence of the mirror had no effect when it occurred at the same time as the presence of dog feces compared to control without mirror and the reproduction of anthropic sounds compared to both controls. Therefore, the sounds themselves were the triggers for the anti-predatory responses.

### Presence of predators

In general, European mink abruptly increased their time in the nest box when they were exposed to predators odors with respect to control. This is a consistent result since the early detection of a predator by indirect cues is in itself the first step in an anti-predatory strategy^24^ being in this case the hiding in their refuge. At the same time, minks increased the time spent in the nest box and time on vigilance during predator odors with respect to control, especially during dog feces exposition. In this sense, it is known that prey are able to recognize volatile compounds from a carnivorous diet^19, 20, 24, 56^. Also, according to Hall et al.^58^ prey perceive the predation risk according to the type of predator (terrestrial or aerial) and its hunting strategy (ambush or persecution) that feeds on them. European mink inhabits areas of high and dense vegetation associated with river borders^44, 72^ which share with predators, so the risk of predation depends, in part, on the type of landscape^73^. Therefore, their behaviors are influenced by a compromise solution between survival and other behaviors such as feeding or reproduction^22–24^. Our research deals with a predatory carnivore which, due to its small size, may also be prey to larger carnivores^58^. In addition, not only they can be predated upon, but they can also be attacked by other dominant carnivores due to interspecific competition for the same resources^47^.

Males spent more time in their nest box during predator feces compared to control probably because they are more patrolling and therefore respond to any threat even if the response time varies. However, females spent more time in their nest box only during dog feces compared to control probably for the reasons given above—about recognition of the carnivorous diet in the feces of predators—they prioritize the response to the most likely risks, because they need to optimize their time and energy more for other activities such as the care of the offspring or the maintenance of their burrow. However, neither sex showed differences in vigilance time during predator odors with respect to control and between them.

In addition, subadults spent more time in their nest box but did not change their time on vigilance when they were exposed to predator feces compared to control but there were not differences between both type of predators. However, adults spent more time in their nest box and increased their time on vigilance during predator feces compared to control, and time spent in the nest box was more pronounced during dog feces treatment. This is probably because younger minks assume any new cues as a risk due to ignorance or inexperience, unlike adults who are able to discriminate more easily by being used to caregiver management.

### Anthropic noises

Anthropic noises were the ones that triggered a more pronounced response with respect to the rest of treatments and control, increasing the time spent in the nest box and decreasing the time on vigilance in the specific case of human voices. As we have already remarked, the sound itself was a cue that triggered an immediate flight response, so European mink hid regardless of the presence/absence of a conspecific. Several researchers found that the rate of encounters of wild animals and humans has increased in locations where the intensity and variety of human leisure activities increased^74^. In such situations, animals could adopt behavior strategies such as hiding^39^. Sounds from anthropic infrastructures or human activities linked to nature are relatively recent threats in their evolutionary history and, until now, less common in their natural habitat. Therefore, it is possible that European mink develop an innate anti-predatory response as an evasive action to an unknown threat source^75, 76^. Despite the very significant differences between acoustic signals in terms of both SPL and their frequency spectra, it is worth noting the lack of differences regarding the amount of time spent by European mink in their nest boxes and, in contrast, differences in vigilance time induced by human voices as it is known that many species consider humans as predators^39^. The fact that the time spent in the nest box increased and the vigilance time decreased in this context could be due to the refuge-vigilance hypothesis by which the preys could perceive the areas of greater coverage (low visibility) as refuge and decrease the time on vigilance. Nest boxes are their main refuge, equivalent to a burrow in the natural habitat^58, 77^.

Both sexes spent more time in the nest box during noise treatments. Males conducted vigilance for longer during traffic noise than females, who abruptly decreased their time spent on vigilance. According to Palazón et al.^78^, road traffic is the main direct cause of mortality of European mink in Spain. These data are also consistent with a research carried out in France which also found road-killed individuals as the main cause of death in this species^79^. Thus, Palazón et al.^80^ highlighted differences on car collisions rate between sexes, finding that the fatal car collisions were higher in males than females at the breeding season. They suggested that this difference may be due to the ecology of European mink, since males travel longer distances and more frequently than females, patrolling and spending more time on foraging^81–83^ which increases males’ likelihood of death across roads. According to our results, more deaths of males than females on the road could be due not only to the dispersive behavior of the males^49^ but also to the fact that females abruptly decrease their vigilance time because road traffic noise pollution itself encourages hiding responses. Males are likely to take more risks and establish a trade-off between crossing the road or hiding, devoting more time on vigilance to avoid collisions. It should be emphasized that the human voices trigger a very strong response in both sexes, probably because, after all, it is an animal vocalization that can be interpreted as a high risk of predation.

In addition, subadult European mink spent more time in their boxes than adults during all treatments, but especially during the acoustic treatments. As regards road traffic noise, it is reasonable to consider that subadults have not yet arrived at their dispersive stage for settling in new territories. In contrast, adult males, already sexually mature, may encounter roads that they have to cross to find females with which to reproduce in the breeding season^49^. Consequently, they must be more careful not to be run over and road traffic SPL could be interpreted as an indicator of distance to the road for them^32, 84^. Finally, it is interesting to note that vigilance response decreased greatly in both age groups during exposure to human voices.

### Additional considerations

In situ conservation of European mink is a difficult task, since it is currently estimated that the Iberian population consists of fewer than 500 individuals^72^. Due to their elusive character, European mink are monitored by trapping and microchip^85^ but, until now, there are no known studies on how individuals respond to potential environmental threats. Our research showed how European mink modified their behavior of hiding and territorial vigilance depending on the environmental factors (presence of competitors, predators, and/or human disturbance cues) but their responses were also modulated by individual factors (sex and age). Therefore, we consider that our results may be of interest for in situ conservation planning and management. In relation to ex situ conservation, in some cases, reintroductions into the natural habitat of captive individuals of endangered species are unsuccessful interventions^80^. An example of this was the 50% mortality (1–1.5 months after release) due to predation of 172 European mink reintroduced in Estonia^48^. A possible explanation is an inadequate anti-predatory response when exposed to unknown predation sources^86^. Although it was also possible that individuals who were isolated from their predators, either throughout their lifetime (ontogeny) or during an evolutionary time (generations), may lose the previously adaptive anti-predator behavior^80, 87^. Therefore, we suggest considering the possibility of carrying out predator training in ex situ conservation centers before reintroductions in the natural habitat, since there are studies based on birds^88^, steppe polecat (*Mustela eversmanii*)^89^, and rufous hare-wallaby (*Lagorchestes hirsitus*)^90^ which supported an improvement in the survival rate in reintroductions. Although in this study minks seem to develop an appropriate innate antidepredatory response, predator and training against other threats can act as environmental enrichment by improving the well-being of European mink whose reproduction is often compromised by the stress to which they may be subjected in captivity^91^. In fact, after the experiment it was the year of greatest reproductive success in the center.

Finally, it is important to note that European mink acted in front of human voices with a predatory behavior much more accentuated than other threats. So, our finding also infer the need of management plans in relation to access and public use of wild and recreation areas; in particular in natural protected areas where anthropic pressures due to recreational and touristic activities follow a continually growing and concerning trend for the last decades^92, 93^. We consider that for future research it is necessary to know whether human disturbances such as noise, among others, and the risk of predation, influence the mink physiologically, which undoubtedly has pathological consequences for a threatened species^36–38^.
